# Possible involvement of peripheral TRP channels in the hydrogen sulfide-induced hyperalgesia in diabetic rats

**DOI:** 10.1186/s12868-018-0483-3

**Published:** 2019-01-03

**Authors:** José Eduardo Roa-Coria, Jorge Baruch Pineda-Farias, Paulino Barragán-Iglesias, Geovanna Nallely Quiñonez-Bastidas, Ángel Zúñiga-Romero, Juan Carlos Huerta-Cruz, Juan Gerardo Reyes-García, Francisco Javier Flores-Murrieta, Vinicio Granados-Soto, Héctor Isaac Rocha-González

**Affiliations:** 1 Sección de Estudios de Posgrado e Investigación, Escuela Superior de Medicina, Instituto Politécnico Nacional, Plan de San Luis y Díaz Mirón s/n, Col. Casco de Santo Tomas, Miguel Hidalgo, 11340 Ciudad de México, Mexico; 2 Neurobiology of Pain Laboratory, Departamento de Farmacobiología, Cinvestav, Unidad Coapa, Calzada de los Tenorios 235, Col. Granjas Coapa, 14330 Ciudad de México, Mexico; 3 Unidad de Investigación en Farmacología, Instituto Nacional de Enfermedades Respiratorias “Ismael Cosio Villegas”, Secretaría de Salud, Calzada de Tlalpan 4502, Col. Sección XVI, Tlalpan, 14080 Ciudad de México, Mexico

**Keywords:** Cystathionine-β-synthase, Hydrogen sulfide, Hyperalgesia, Painful diabetic peripheral neuropathy, Streptozotocin-induced diabetes, Transient receptor potential channels

## Abstract

**Background:**

Peripheral diabetic neuropathy can be painful and its symptoms include hyperalgesia, allodynia and spontaneous pain. Hydrogen sulfide (H_2_S) is involved in diabetes-induced hyperalgesia and allodynia. However, the molecular target through which H_2_S induces hyperalgesia in diabetic animals is unclear. The aim of this study was to determine the possible involvement of transient receptor potential (TRP) channels in H_2_S-induced hyperalgesia in diabetic rats.

**Results:**

Streptozotocin (STZ) injection produced hyperglycemia in rats. Intraplantar injection of NaHS (an exogenous donor of H_2_S, 3–100 µg/paw) induced hyperalgesia, in a time-dependent manner, in formalin-treated diabetic rats. NaHS-induced hyperalgesia was partially prevented by local intraplantar injection of capsazepine (0.3–3 µg/paw), HC-030031 (100–316 µg/paw) and SKF-96365 (10–30 µg/paw) blockers, at 21 days post-STZ injection. At the doses used, these blockers did not modify formalin-induced nociception. Moreover, capsazepine (0.3–30 µg/paw), HC-030031 (100–1000 µg/paw) and SKF-96365 (10–100 µg/paw) reduced formalin-induced nociception in diabetic rats. Contralateral injection of the highest doses used did not modify formalin-induced flinching behavior. Hyperglycemia, at 21 days, also increased protein expression of cystathionine-β-synthase enzyme (CBS) and TRPC6, but not TRPA1 nor TRPV1, channels in dorsal root ganglia (DRG). Repeated injection of NaHS enhanced CBS and TRPC6 expression, but hydroxylamine (HA) prevented the STZ-induced increase of CBS protein. In addition, daily administration of SKF-96365 diminished TRPC6 protein expression, whereas NaHS partially prevented the decrease of SKF-96365-induced TRPC6 expression. Concordantly, daily intraplantar injection of NaHS enhanced, and HA prevented STZ-induced intraepidermal fiber loss, respectively. CBS was expressed in small- and medium-sized cells of DRG and co-localized with TRPV1, TRPA1 and TRPC6 in IB4-positive neurons.

**Conclusions:**

Our data suggest that H_2_S leads to hyperalgesia in diabetic rats through activation of TRPV1, TRPA1 and TRPC channels and, subsequent intraepidermal fibers loss. CBS enzyme inhibitors or TRP-channel blockers could be useful for treatment of painful diabetic neuropathy.

**Electronic supplementary material:**

The online version of this article (10.1186/s12868-018-0483-3) contains supplementary material, which is available to authorized users.

## Background

Diabetes is a chronic disease that occurs when the pancreas does not produce enough insulin or when the body cannot effectively use insulin, leading to raised glucose levels in the blood. In North America and Caribbean region there were 45.9 million of people with diabetes in 2017, and it has been estimated that there will be 62.2 million of people with diabetes in 2045 [1]. The long-term hyperglycemia is associated with damage to the body and failure of various organs and tissues. Diabetic peripheral neuropathy is the most common and debilitating complication of diabetes, which is characterized by symptoms of peripheral nerve dysfunction as hyperalgesia and allodynia [2]. The treatment for painful diabetic peripheral neuropathy includes glycemic control and symptomatic pain management. Food and Drug Administration has approved only duloxetine, pregabalin and tapentadol for the treatment of painful diabetic neuropathy, but they have low efficacy and are able to cause serious side effects [3, 4]. The lack of an adequate pharmacological treatment supports the need to clarify better the diabetes-associated painful peripheral neuropathy pathogenesis to relieve this complication.

Hydrogen sulfide (H_2_S), a gasotransmitter, is mainly synthesized from l-cysteine by cystathionin-β-synthase (CBS), cystathionin-γ-lyase (CSE) and 3-mercaptopyruvate sulfurtransferase in mammals [5, 6]. At physiological concentrations, H_2_S appears to have a cytoprotective effect. However, its excessive production seems to contribute to the pathogenesis of painful disorders, septic shock and cerebral stroke, among others [7, 8].

Most of literature indicates that H_2_S has a pronociceptive effect in somatic, visceral and neuropathic pain processing [9], although there is evidence suggesting an antinociceptive role of H_2_S in neuropathic pain [10]. In one of the neuropathic pain studies, authors reported that the administration of CSE inhibitors decreased hyperalgesia and allodynia evoked by the right L5 spinal nerve transection in rats [11]. In addition, intraplantar application of NaHS (a donor of H_2_S) leads to hyperalgesia in sham-operated rats, but not in rats subjected to genetic silencing of Ca_V_3.2 T-type Ca^2+^ channels, indicating that CSE-derived endogenous H_2_S contributes to the maintenance of neuropathic pain through activation of Ca_V_3.2 T-type Ca^2+^ channels. In line with above results, the CSE-H_2_S-Ca_V_3.2 T-type Ca^2+^ channels pathway is suggested to mediate the paclitaxel-induced neuropathic hyperalgesia [12]. In fact, it has been demonstrated that there are redox sites in the T-type Ca^2+^ channels where H_2_S may be exerting its peripheral sensitization effect on nociceptors [13, 14]. A recent study shows that peripheral nerve injury increases CSE expression in dorsal root ganglia (DRG). Furthermore, conditional knockout mice lacking CSE in sensory neurons has a pain threshold similar to wild type mice, suggesting the existence of a compensatory mechanism by other H_2_S-producing enzymes [15].

In this regard, Wang and colleagues reported that CBS and P2X3, but not CSE, were up-regulated in a model of lumbar disc herniation. Moreover, the systemic administration of a CBS inhibitor reversed the over-expression and function of P2X3 receptors, indicating the CBS-H_2_S-P2X3 signaling participates in the lumbar disc herniation-induced hypersensitivity [16]. Similarly, it has been suggested that CBS-H_2_S pathway participates in the chronic constriction injury-induced allodynia through the activation of NF-κβ(p65), ERK1/2 and CREB pathways [17].

Our previous study suggests that H_2_S is involved in diabetes-induced hyperalgesia and allodynia. However, the molecular targets through which H_2_S induces painful disorders in diabetic neuropathy remains unclear [18]. It has been postulated that H_2_S could modify the activity of transient receptor potential (TRP) channels [19]. TRP channels are cation channels permeable to both monovalent and divalent cations as Na^+^ or Ca^2+^. They are expressed in sensory neurons [20, 21] and are involved in the modulation of diabetes-induced painful peripheral neuropathy [22–24]. In addition, there is evidence that H_2_S activates TRPA1 and TRPV1 channels to produce visceral pain [25, 26]. Based on the above considerations, the current study was undertaken to investigate the possible participation of peripheral TRPV1, TRPA1 and TRPC channels in H_2_S-induced hyperalgesia in diabetic rats.

## Results

### Streptozotocin-induced hyperglycemia and increased nociceptive behavior in the formalin test

**Fig. 1:**
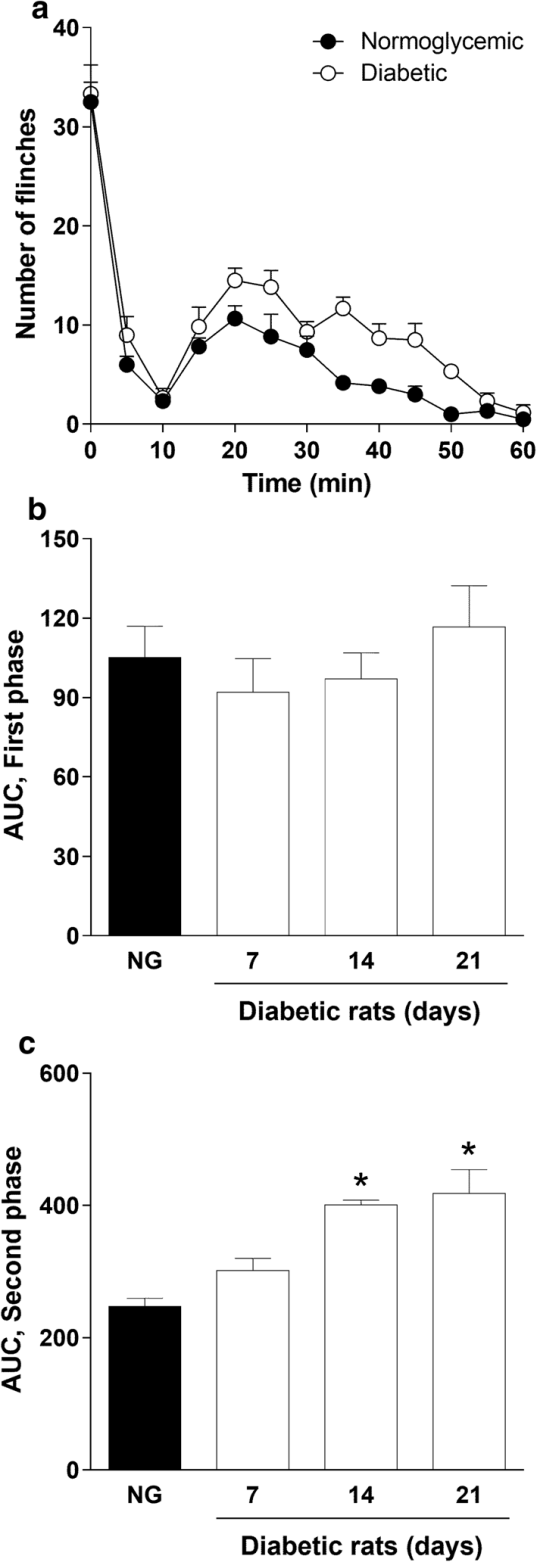
Diabetes-induced nociceptive behavior in the rat formalin test. **a** Temporal courses of the nociceptive behavior observed after injection of 0.5% formalin into the dorsal hind paw of normoglycemic and diabetic rats 21 days after streptozotocin injection. **b**, **c** Nociceptive behavior induced by 0.5% formalin during first and second phase in normoglycemic (NG) and diabetic rats 7, 14 and 21 days after streptozotocin injection. Data are expressed as mean ± SEM for 6 animals. *Significantly different with respect to the normoglycemic group (NG), determined by one-way ANOVA followed by the Dunnett’s test with a *P* < 0.05

Intraperitoneal injection of streptozotocin, but not vehicle, produced hyperglycemia in non-fasted rats after 21 days. The blood glucose level measured in these rats was 85.3 ± 5.3 mg/dL and 81.7 ± 3.2 mg/dL before vehicle or streptozotocin injection and 79.2 ± 7.1 mg/dL and 398.9 ± 8.5 mg/dL 3 weeks after vehicle or streptozotocin injection, respectively. In addition, hyperglycemic rats showed polyphagia, polydipsia, polyuria and body weight loss (data not shown). Formalin injection at 0.5% in normoglycemic and diabetic rats induced a typical biphasic paw flinching behavior. First phase began immediately after formalin administration and had a duration of about 10 min. Second phase started about 15 min after formalin administration, had a maximum between 20 and 25 min, and lasted about 45 min (Fig. 1a). Diabetic rats displayed a greater flinching behavior compared to normoglycemic rats during the second phase, but not in the first phase, after 14 and 21 days of the streptozotocin injection (Fig. 1b, c).

### Effect of H_2_S on formalin-induced nociception in diabetic rats

**Fig. 2:**
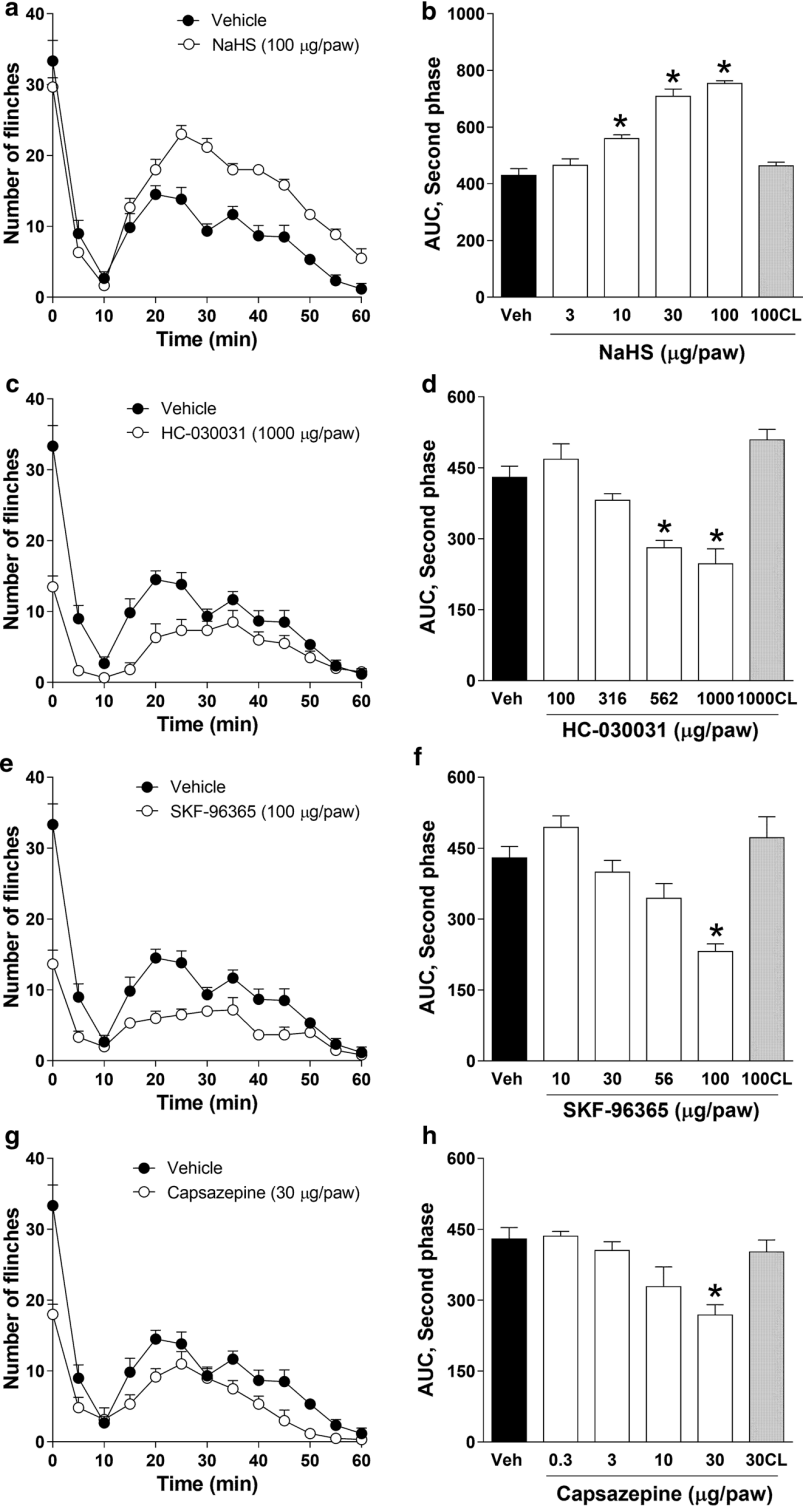
Effect of H_2_S donor and TRP channel antagonists on 0.5% formalin-induced nociceptive behavior in diabetic rats. **a**, **b** NaHS-induced hyperalgesia (a donor of H_2_S; 3–100 μg/paw, s.c.) in diabetic rats subjected to the formalin test. Antinociceptive effect observed after subcutaneous administration of vehicle (Veh) or increasing doses of **c**, **d** HC-030031 (TRPA1 antagonist; 100–1000 μg/paw), **e**, **f** SKF-96365 (TRPC antagonist; 10–100 µg/paw) or **g**, **h** capsazepine (TRPV1 antagonist; 0.3–30 μg/paw, s.c.) during the second phase of the 0.5% formalin test in diabetic rats. Data are expressed as mean ± SEM of 6 animals. *Significantly different with respect to the vehicle (Veh), determined by one-way ANOVA followed by Dunnett’s test with a *P* < 0.05. *CL* contralateral

Ipsilateral peripheral pre-treatment (− 10 min) with NaHS (100 µg/paw) significantly (*P* < 0.05) increased 0.5% formalin-induced flinching behavior during the second phase (Fig. 1a–c), but not during the first phase (Fig. 1a, b), in diabetic rats. NaHS-induced hyperalgesia reached its maximum effect 3 weeks after streptozotocin injection in the 0.5% formalin test (Fig. 1a–c). In addition, NaHS (3–100 µg/paw) enhanced in a dose-dependent manner formalin-induced flinching behavior (*P* < 0.05) during the second phase (Fig. 2a, b), but not during the first phase of the formalin test, on day 21 after streptozotocin injection. In contrast, contralateral peripheral pre-treatment with NaHS (100 µg/paw) did not modify 0.5% formalin-induced nociceptive behavior (Fig. 2b).

### Effect of TRP channel blockers on diabetes-induced nociception

Ipsilateral, but not contralateral, peripheral pre-treatment (−20 min) with the TRPA1, TRPC and TRPV1 channel blockers HC-030031 (100–1000 µg/paw, Fig. 2c, d) SKF-96365 (10–100 µg/paw, Fig. 2e, f) and capsazepine (0.3–30 µg/paw, Fig. 2g, h), respectively, reduced (*P* < 0.05) 0.5% formalin-induced nociceptive behavior during the first (data not shown) and second phases (Fig. 2a–h) of the test, in diabetic rats at 21 days after streptozotocin treatment.

### Effect of TRP channel blockers on NaHS-induced hyperalgesia in diabetic rats

**Fig. 3:**
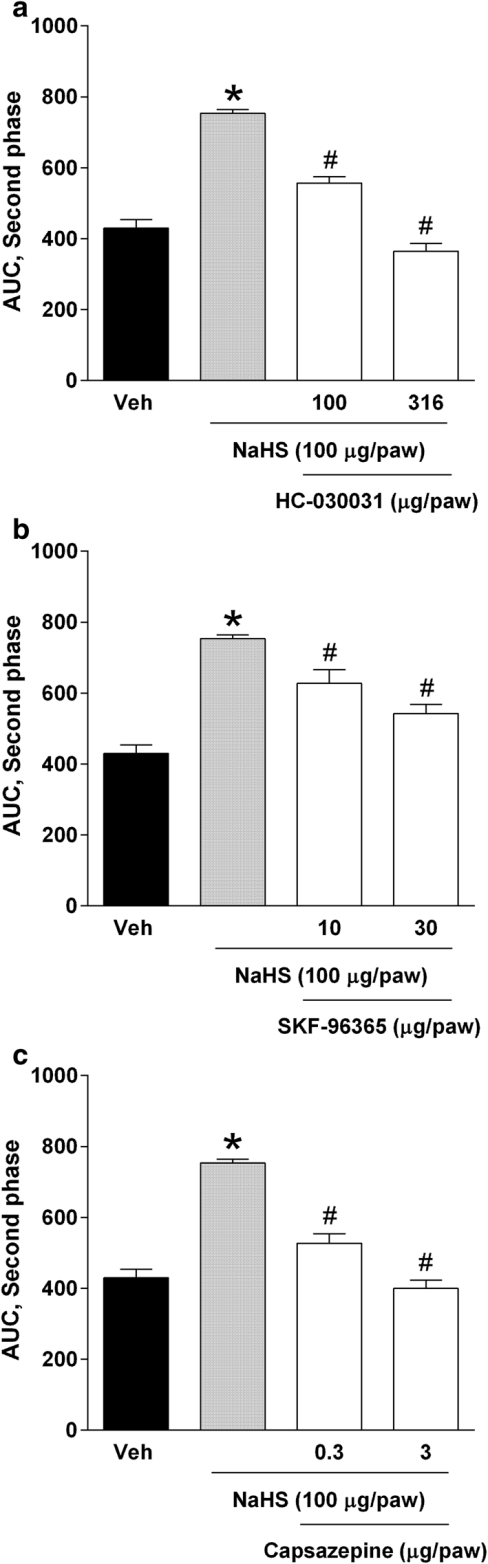
Effect of the local peripheral administration of **a** HC-030031 (100, 316 μg/paw), **b** SKF-96365 (10, 30 μg/paw) and **c** capsazepine (0.3, 3 μg/paw) on NaHS-induced pronociception (100 μg/paw) in diabetic rats submitted to the 0.5% formalin test. Bars are the mean ± SEM of 6 animals. *Significantly different with respect to vehicle (*P* < 0.05) and ^**#**^significantly different from the NaHS group, as determined by one-way ANOVA followed by Tukey’s test

Local peripheral injection of capsazepine (0.3 and 3 µg/paw, Fig. 3a), HC-030031 (100 and 316 µg/paw, Fig. 3b) and SKF-96365 (10 and 30 µg/paw, Fig. 3c) prevented in a dose-dependent manner (P < 0.05) the hyperalgesic effect of NaHS in (3 weeks) diabetic rats in the second phase of the 0.5% formalin test.

### Streptozotocin enhances chemical nociception and, protein expression of CBS, and TRPC6, but not TRPA1 nor TRPV1, in DRG

**Fig. 4:**
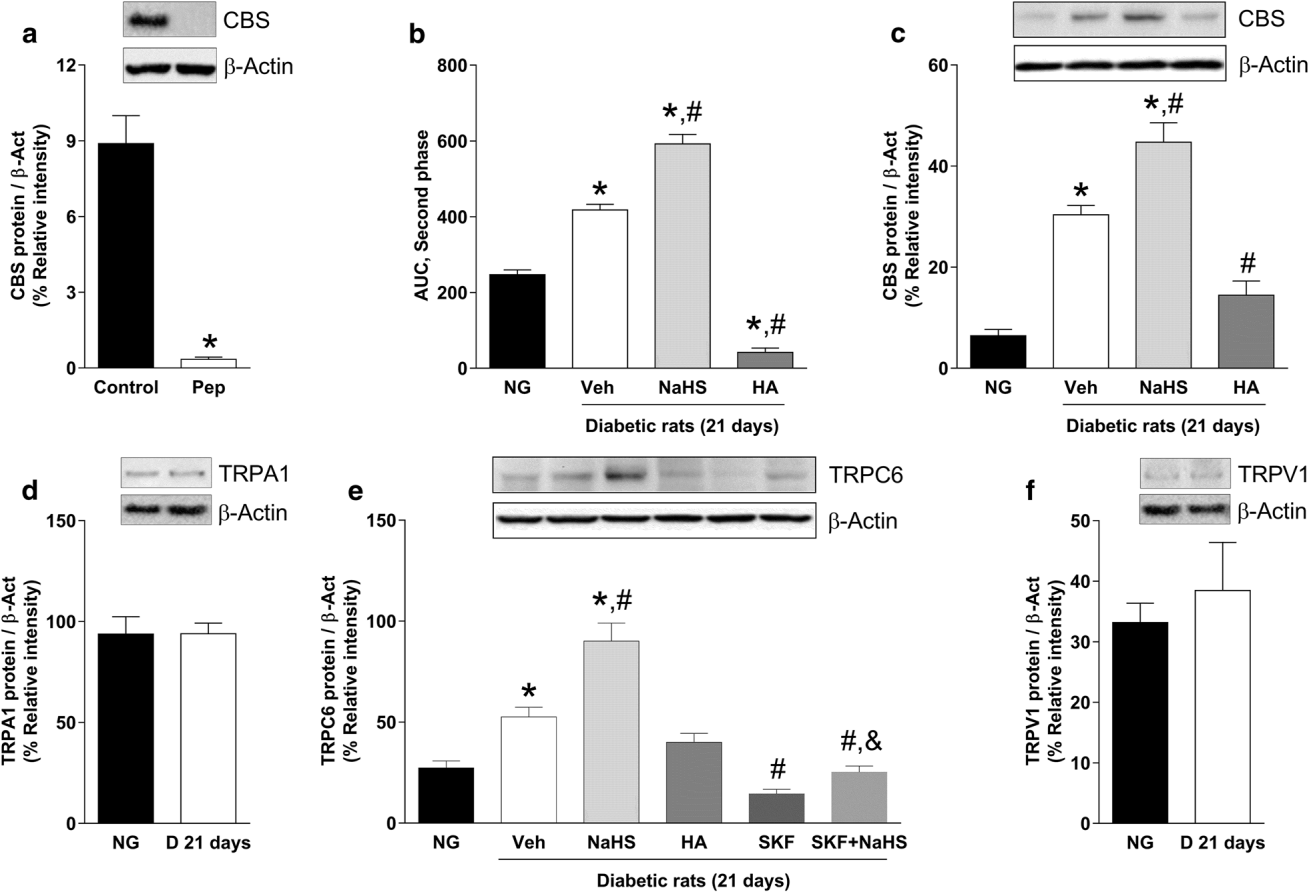
Nociceptive behavior and CBS, TRPA1, TRPC6 and TRPV1 protein expression of the dorsal root ganglia obtained from normoglycemic and diabetic rats at 21 days post-streptozotocin injection. **a** Protein expression of CBS (control) or CBS + control peptide (Pep) in normoglycemic rats. **b** Nociceptive behavior and **c** CBS protein expression obtained in normoglycemic rats (NG) and diabetic rats (D) submitted to subcutaneous administration of vehicle (Veh), NaHS (100 μg/paw) or hydroxylamine (HA; 0.1 μg/paw) during 21 days. **d** TRPA1 protein expression in normoglycemic and diabetic rats 21 days after streptozotocin injection. **e** TRPC6 channel protein expression observed in normoglycemic rats (NG) and diabetic rats treated with vehicle (Veh), NaHS (100 μg/paw), hydroxylamine (HA; 0.1 μg/paw), SKF-96365 (SKF; 100 μg/paw, s.c.) or SKF + NaHS (100 μg/paw + 100 μg/paw, respectively) during 21 days. **f** TRPV1 protein expression in normoglycemic and diabetic rats 21 days after streptozotocin injection. Data were normalized against β-actin and are expressed as the mean ± SEM of 5–6 animals. *Significantly different from control or normoglycemic (NG) group, ^#^significantly different from vehicle (Veh, diabetic rats) group and ^&^significantly different from SKF-96365 (SKF) group (*P* < 0.05) determined by one-way ANOVA followed by the Student–Newman–Keuls’ test

Using western blotting, bands corresponding to the CBS enzyme or TRPV1, TRPA1 and TRPC6 channels were found in the ipsilateral L4-L6 DRG on normoglycemic and (3 weeks) diabetic rats (Fig. 4). Bands disappeared when TRPV1, TRPA1 or TRPC6 primary antibodies were omitted (data not shown). In addition, no signal was observed when the CBS antibody was pre-incubated with its respective control antigenic peptide (Fig. 4a). Interestingly, streptozotocin administration increased significantly 0.5% formalin-induced flinching behavior (Fig. 4b) and protein expression of the CBS enzyme (Fig. 4c) and TRPC6 (Fig. 4e), but not TRPA1 (Fig. 4d) nor TRPV1 (Fig. 4f), channels in L4-L6 DRG.

### Effect of NaHS and hydroxylamine on streptozotocin-induced enhanced expression of CBS enzyme and TRPC6 channel proteins in DRG and, formalin-induced chemical nociception

Repeated administration of NaHS (100 µg/paw, s.c.) for 21 days after the streptozotocin injection augmented (*P* < 0.05) chemical nociception (Fig. 4b), as well as streptozotocin-induced enhanced expression of the H_2_S-producing enzyme CBS (Fig. 4c) and TRPC6 channel (Fig. 4e), proteins. On the contrary, daily local peripheral administration of hydroxylamine (0.1 µg/paw, s.c., 21 days), decreased (*P* < 0.05) streptozotocin-induced enhanced chemical nociception (Fig. 4b) and expression of the CBS enzyme (Fig. 4e).

### Effect of SKF-96365 on streptozotocin-induced enhanced expression of TRPC6 channel protein in DRG

Subcutaneous SKF-96365 administration during 21 days (100 µg/paw) decreased streptozotocin-induced enhanced expression of the TRPC6 channel. Furthermore, NaHS (100 µg/paw, s.c.) partially prevented the SKF-96365-induced down-regulation of the TRPC6 protein expression when both drugs were co-administered during 3 weeks (Fig. 4e).

### CBS is expressed in IB4-positive DRG neurons and it co-localizes with TRPV1, TRPA1 and TRPC6

**Fig. 5:**
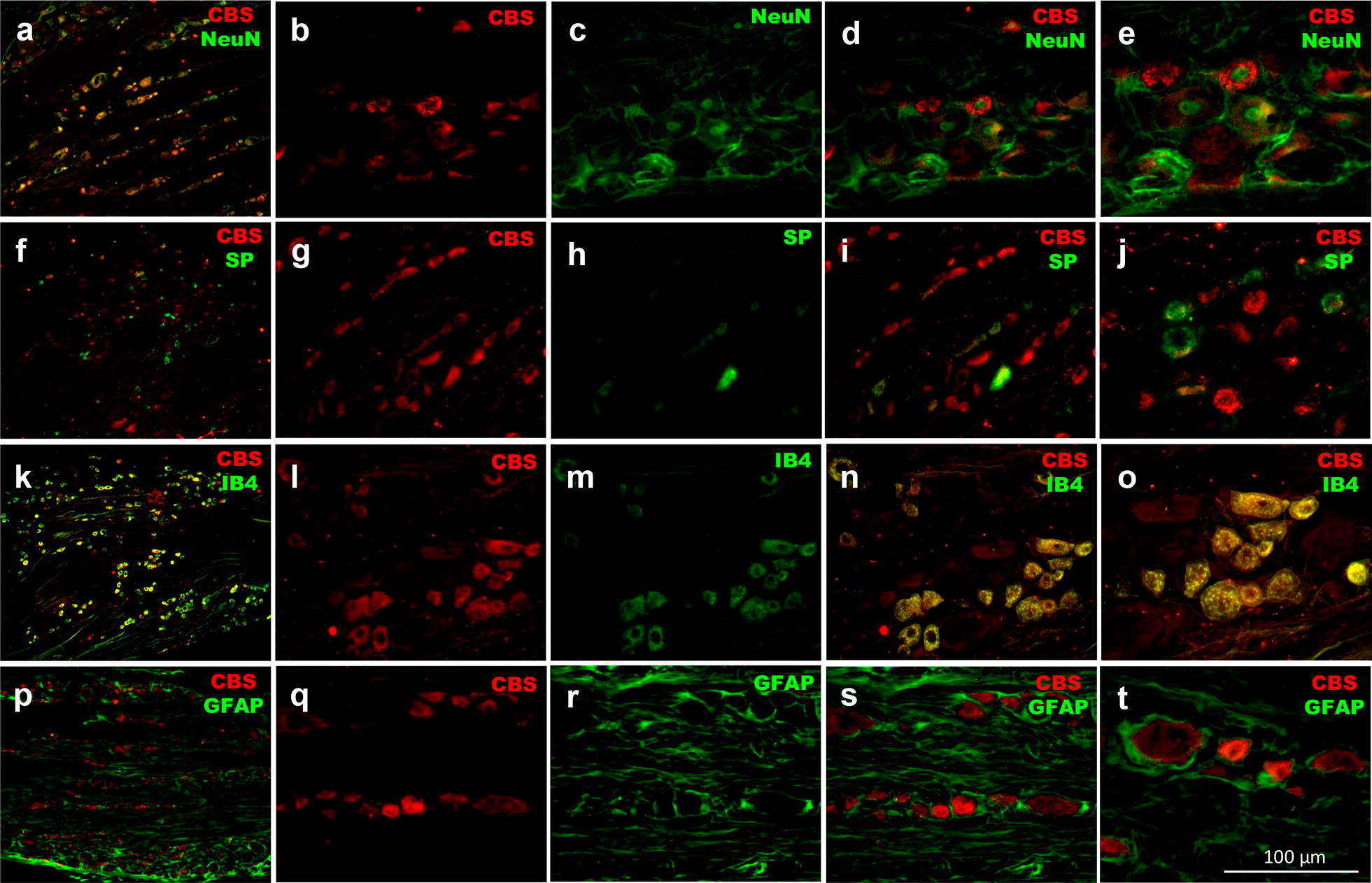
Immunolocalization of cystathionin-β-synthase enzyme (CBS, red) with **a**–**e** neuronal nuclear antigen (NeuN)-, **f**–**j** substance P (SP)-, **k**–**o** isolectin B4 (IB4)- and **p**–**t** glial fibrillary acidic protein (GFAP)-positive dorsal root ganglion neurons of diabetic rats (green). **a**, **f**, **k** and **p** show a representative 16 µm-slice from dorsal root ganglia. **b**, **g**, **l** and **q** show a representative CBS staining with Cy3 from dorsal root neurons, whereas **c**, **h**, **m** and **r** show NeuN, SP, IB4 and GFAP representative staining with Cy2 in dorsal root neurons, respectively. **d**, **i**, **n** and **s** show the merged image from Cy3 and Cy2 signals, the overlapping between CBS and neuronal markers is shown in a yellow-orange color. **e**, **j**, **o** and **t** show a magnification of **d**, **i**, **n** and **s**, respectively

CBS enzyme co-localized with the neuronal marker NeuN suggesting that it is present in DRG neurons (Fig. 5A–E). Moreover, CBS co-localized with 3.4 ± 0.4% of SP-positive neurons (Fig. 5F–J) and with 96.2 ± 4.2% of IB4-positive DRG neurons (Fig. 5K–O). In contrast, CBS did not co-localize with GFAP suggesting that is not present in astrocytes (Fig. 5P–T).

**Fig. 6:**
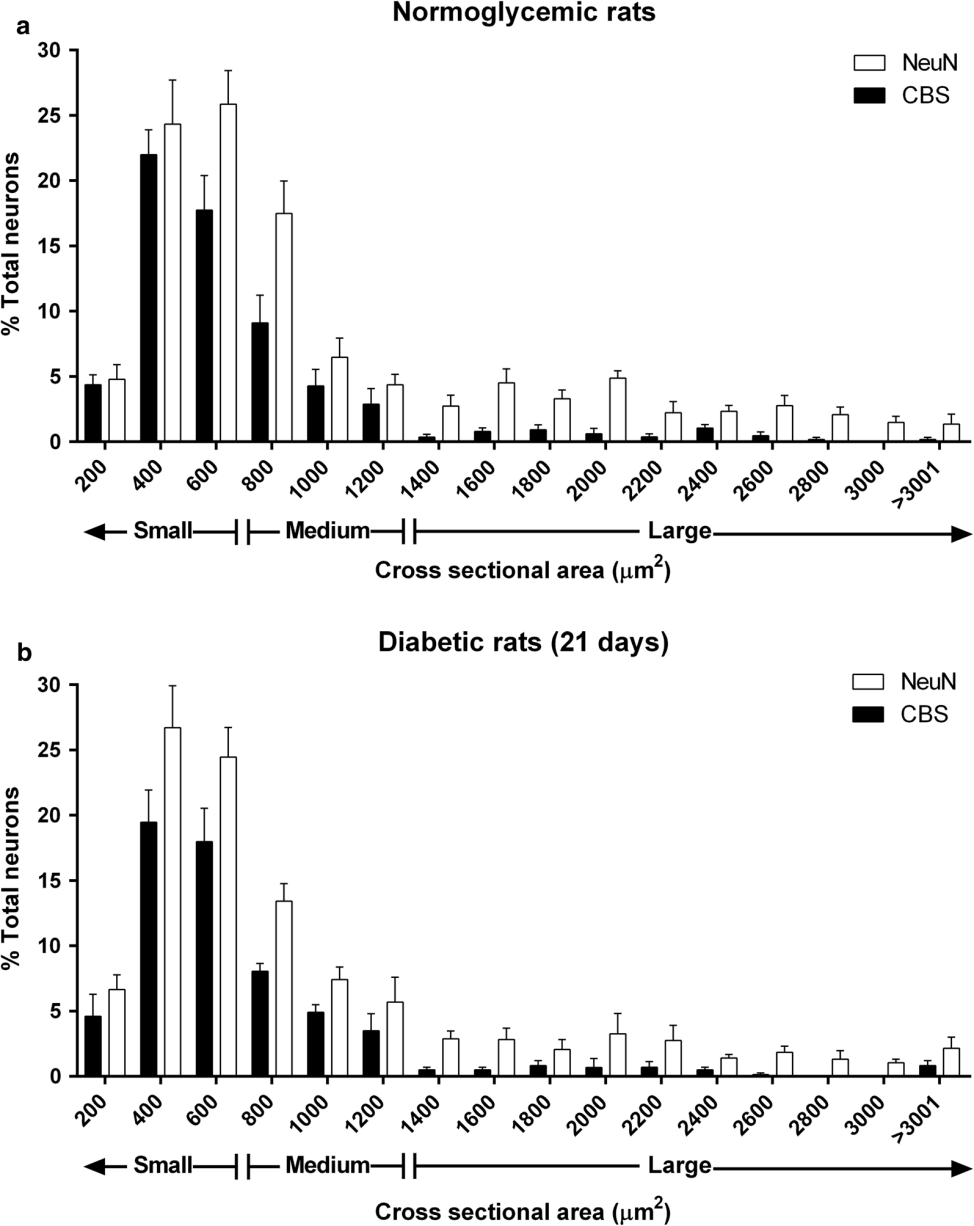
Distribution histograms that show CBS expression in dorsal root neurons of **a** normoglycemic and **b** diabetic rats 21 days after administration with streptozotocin. The graphs represent the number of CBS-positive neurons (black bars) or the number of NeuN-positive neurons (white bars) stratified according the neuron cross sectional area in μm^2^. Data are expressed as the average percentage ± SEM of 10–12 longitudinal cross-sections from three rats. There is no statistical difference between CBS-positive neurons of normoglycemic and diabetic rats (*P* > 0.05), as determined by two-way ANOVA

**Fig. 7:**
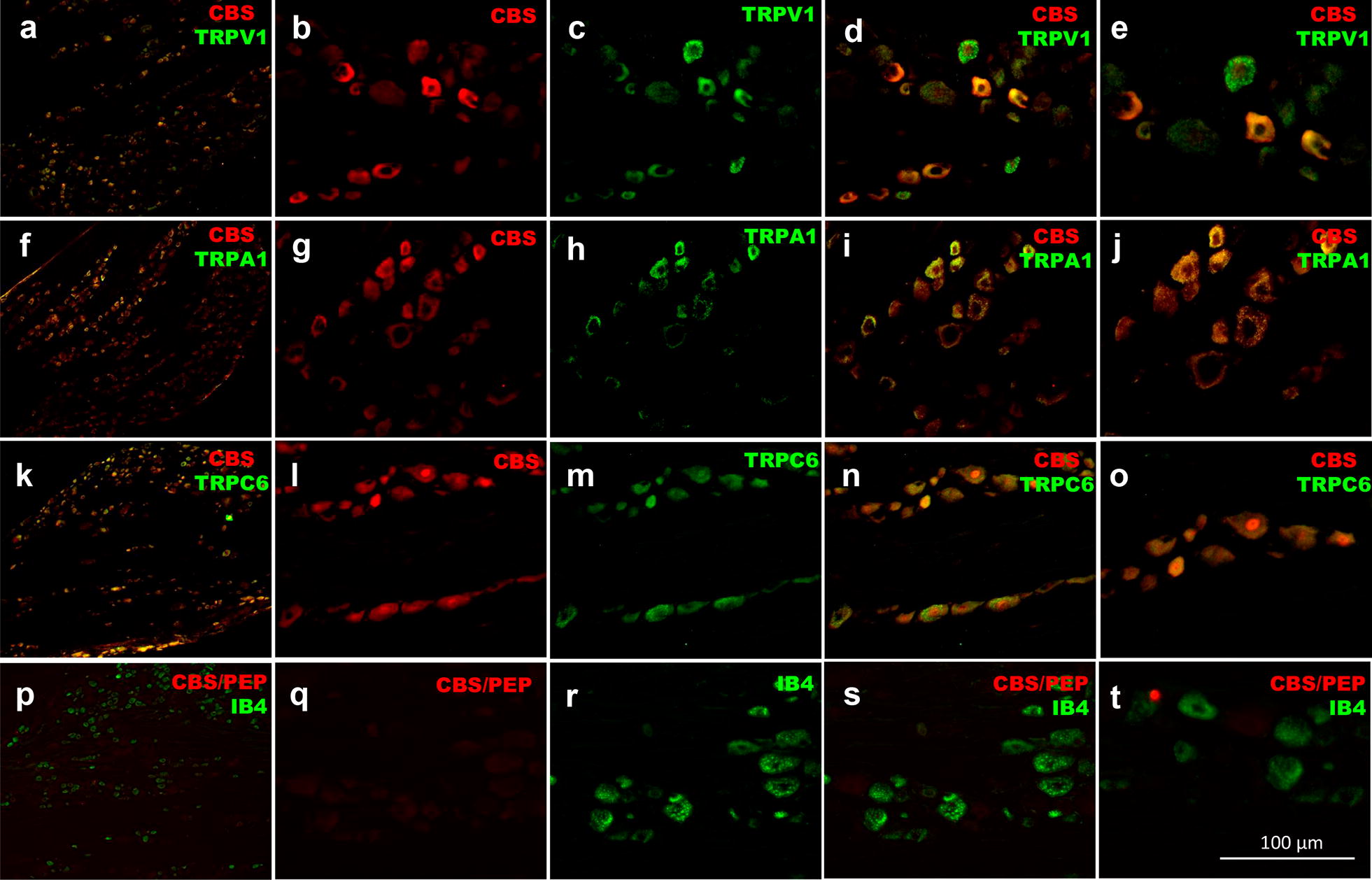
Immunolocalization of cystathionin-β-synthase enzyme (CBS, red) in **a**–**e** TRPV1-, **f**–**j** TRPA1- and **k**–**o** TRPC-positive dorsal root ganglion neurons of diabetic rats (green). **a**, **f** and **k** show a representative 16 µm-slice from dorsal root ganglia. **b**, **g** and **l** show a representative CBS staining with Cy3 from dorsal root neurons, whereas **c**, **h** and **m** show TRPV1, TRPA1 and TRPC6 representative staining with Cy2 in dorsal root neurons, respectively. **d**, **i** and **n** show the merged image from Cy3 and Cy2 signals, the overlapping between CBS and TRP channels is shown in a yellow-orange color. **e**, **j** and **o** show a magnification of **d**, **i** and **n**, respectively

CBS was found mainly in small and medium size DRG neurons (Fig. 6). The pattern of distribution of CBS in DRG neurons of normoglycemic rats was similar to that observed in diabetic animals (21 days). CBS co-localized with 87.5 ± 6.9% of TRPV1- (Fig. 7A–E), 94.8 ± 3.9% of TRPA1- (Fig. 7F–J) and 91.6 ± 6.3% of TRPC6-positive (Fig. 7K–O) neurons in diabetic rats. No signal was observed when the CBS antibody was pre-incubated with its respective control antigenic peptide (Fig. 7P–T), or when TRPA1, TRPV1 or TRPC6 primary antibodies were omitted (data not shown).

### Effect of NaHS and hydroxylamine on the loss of intraepidermal nerve fibers in diabetic rats

**Fig. 8:**
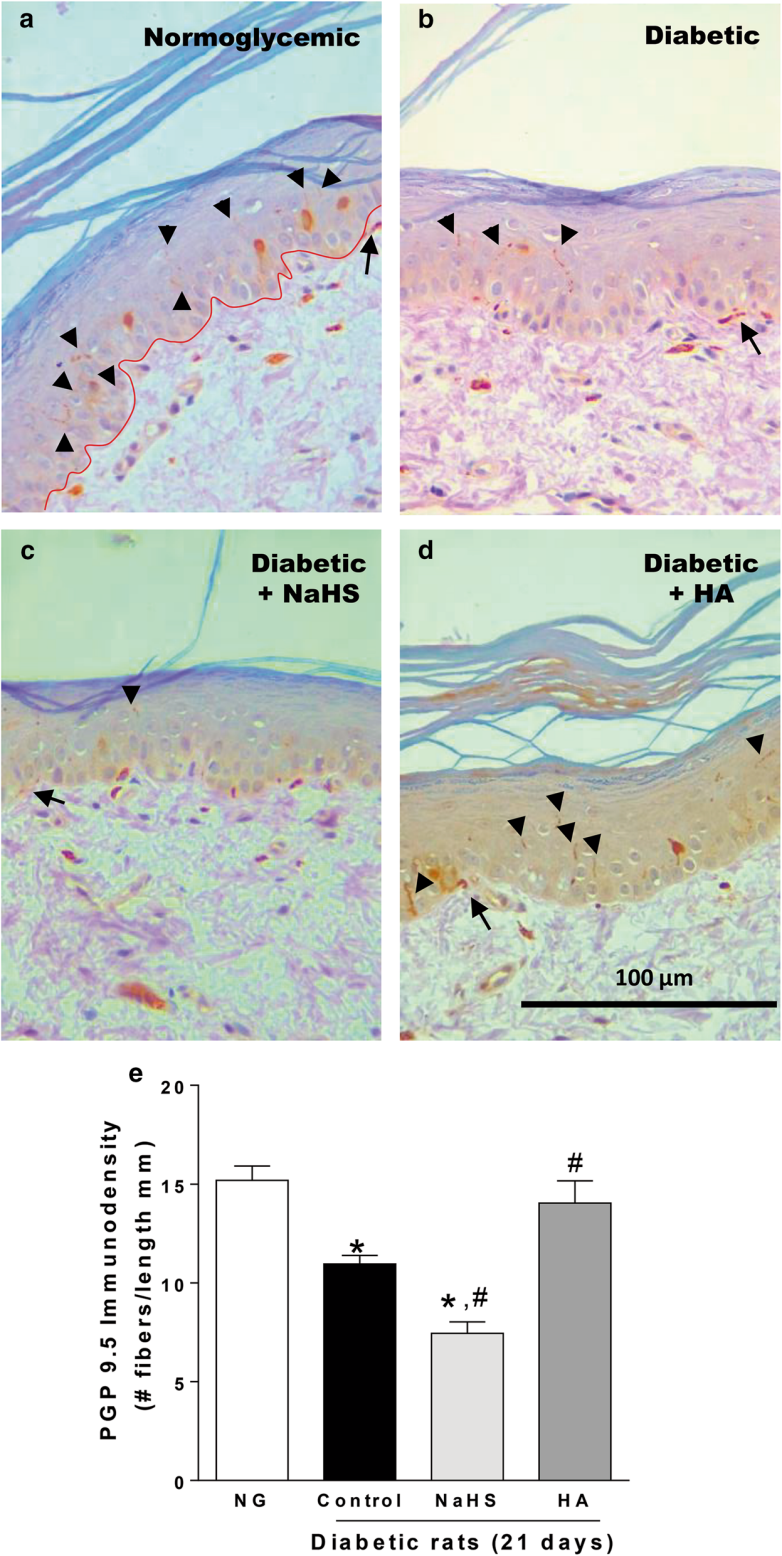
Representative images for skin sections immunostained with PGP 9.5 to reveal the innervation of intraepidermal (black arrowheads) and subepidermal nerve fibers (black arrows) at × 40 magnification. **a** Sections in normoglycemic rats show abundant epidermal nerve profiles, **b** whereas sections in diabetic rats reveal a decrease in nerve fibers. **c** Daily NaHS treatment (a donor of H_2_S; 100 μg/paw, 21 days, s.c.) enhances diabetes-induced nerve fiber loss. On the contrary, **d** daily subcutaneous hydroxylamine (a CBS enzyme inhibitor [HA]; 0.1 μg/paw, 21 days) partially reversed the diabetes-induced nerve fiber loss. Bars are represented as the mean of the number of nerve fibers counted per mm of epidermis ± SEM of 5 animals for each treatment. *Significantly different from normoglycemic (NG) group and ^#^significantly different from control (diabetic rats) group (*P* < 0.05) determined by one-way ANOVA followed by the Student–Newman–Keuls’ test. Red line separate epidermis to dermis

Treatment with streptozotocin, not only produced hyperglycemia and hyperalgesia (Fig. 1) in diabetic rats of 21 days, but also resulted in loss of nerve fibers innervating the skin from the hindpaw with respect to normoglycemic animals (Fig. 8a, b). Daily subcutaneous administration of NaHS (100 µg/paw) enhanced streptozotocin-induced epidermal nerve fibers loss (Fig. 8c), while hydroxylamine (0.1 µg/paw) decreased the loss of epidermal nerve fibers induced by streptozotocin (Fig. 8d, e).

## Discussion

The current study suggests for the first time the possible participation of TRP channels as molecular targets of H_2_S to induce painful peripheral neuropathy in diabetic rats. In our study, peripheral injection of NaHS, a donor of H_2_S, led to a time-dependent and dose-dependent hyperalgesic effect in the formalin test in diabetic rats. Our study matches with previous works showing the pronociceptive effect of peripheral H_2_S in neuropathic pain models, such as L5 spinal nerve axotomy [11] or diabetes-induced peripheral neuropathy [18]. In fact, this effect is in line with previous studies that show higher levels of H_2_S in the rat hindpaw tissue after a noxious stimulus such as paclitaxel-evoked hyperalgesia [12] or formalin injection [27]. Paradoxically, in our previous study, the H_2_S levels did not change in the paw tissue during the diabetes development [18], suggesting that the H_2_S-induced neuropathic hyperalgesia in diabetic rats could be explained through mechanisms such as up-regulation or activation/sensitization of other proteins.

Currently, several mechanisms have been proposed in an attempt to explain the pronociceptive effect of H_2_S. The evidence points out in somatic, visceral and neuropathic pain models that H_2_S exerts its pronociceptive effect through the activation of Ca_V_3.2 T-type Ca^2+^ channels [9, 11, 12, 25, 28–30]. In this regard, H_2_S may be acting directly on Ca_V_3.2 T-type Ca^2+^ channel redox sites since l-cysteine-enhanced T-current in rat sensory neurons and this effect was partially blocked by mibefradil and the oxidizing agent dinitrothiocyanobenzene [13]. In addition, H_2_S seems to sensitize P2X3 channels to produce persistent pain in a lumbar disc herniation model [16], it suppresses sustained potassium channel currents [31, 32] and increases sodium current density [33], in trigeminal and DRG neurons, respectively. Likewise, H_2_S enhances phosphorylation and expression of NMDA channels in somatic and visceral pain [34, 35], up-regulates TRPV1 channels [26] and activates TRPA1 channels in somatic and visceral pain models [25, 36–38]. However, the evidence about the molecular mechanism targeted by H_2_S to induce painful diabetic peripheral neuropathy remains to be elucidated.

In the current study, we hypothesized that H_2_S would act on TRP channels to induce hyperalgesia in diabetic rats. In this regard, local peripheral injection of the capsazepine, HC-030031 and SKF-976365 blockers decreased in a dose dependent manner the diabetes-induced peripheral nociception in the formalin test. Our pharmacological studies suggest that TRPV1, TRPA1 and TRPC channels participate in the pronociceptive effect of H_2_S in diabetic rats. In support of this, we found that NaHS-induced hyperalgesia in diabetic rats was partially prevented by increasing doses of capsazepine, HC-030031 and SKF-96365 blockers. The current study agrees with previous observations showing that TRPV1 [39, 40] and TRPA1 [41] channels play an important pronociceptive role in the painful diabetic neuropathy and extends these observations to TRPC channels. Notwithstanding, these findings should be interpreted with caution since capsazepine also antagonizes voltage-activated calcium channels [42], acethylcholine receptors [43], and hyperpolarizing-activated cyclic nucleotide-gated channels [44], whereas SKF-96365 is also an inhibitor of voltage-gated and receptor-mediated Ca^2+^ entry [45, 46].

We found that experimental diabetes increased TRPC6, but not TRPV1 nor TRPA1, protein expression in DRG. In addition, daily subcutaneous NaHS treatment enhanced streptozotocin-induced TRPC6 protein expression. Interestingly, CBS enzyme highly co-localized with TRPA1, TRPV1 and TRPC6 channels in small and medium IB4-positive cells from DRG. In agreement with the pronociceptive effect of H_2_S through TRP channels. The literature points out that NaHS increases intracellular Ca^2+^ in TRPA^+/+^ mice, but not in TRPA1^−/−^ mice [36] and that the NaHS-induced pronociceptive effect is abolished by TRPA1 inhibitors or by silencing with antisense oligodeoxynucleotides of TRPA1 channels [25, 38]. Furthermore, it has been postulated that the reaction of H_2_S and NO produces HNO^-^ anion, which in turn, activates TRPA1 channels via formation of amino-terminal disulphide bonds [47]. In our study, since TRPA1 blocker was able to reduce NaHS-induced hyperalgesia, but TRPA1 protein expression was not modified during diabetes development, our data suggest that H_2_S could activate TRPA1 channels to induce nociception in diabetic rats, via an indirect mechanism, more than a direct mechanism.

On the other hand, the experiments designed to demonstrate the direct activation of TRPV1 receptors by H_2_S or polysulfides have been negative. In this regard, NaHS or Na_2_S are not able to induce Ca^2+^ influx into the chinese hamster ovary cells expressing TRPV1 receptors [36], nor the TRPV1 blocker BCTC decreases the Na_2_S_3_-induced intercellular Ca^2+^ increases in sensory neurons [38]. Notwithstanding, there is evidence to indicate that H_2_S indirectly sensitizes TRPV1 channels. In this respect, the expression of TRPV1 channels is up-regulated by H_2_S formed by the CBS enzyme in visceral pain [26]. Furthermore, H_2_S seems to promote the development of neuropathic pain through activation of ERK1/2, NF-κβ(p65) and CREB [17], which in turn up-regulate the expression of several pronociceptive cytokines, enzymes, receptors and ionic channels [48, 49]. In our study, TRPV1 protein expression was not up-regulated during diabetes development. Differences between Zhu and colleagues [26] and our study in the TRPV1 modulation by H_2_S may be attributed to pain model (visceral pain versus neuropathic pain). Another mechanism proposed to explain the sensitization of channels by H_2_S is protein phosphorylation. In this regard, it has been reported that overexpression of CBS leads to phosphorylation of NMDA receptors via up-regulation of PKCγ [36], and possibly the phosphorylation of TRPV1 for the same mechanism [50].

We observed that TRPC blocker reduced H_2_S-induced hyperalgesia in diabetic rats, TRPC6 protein was overexpressed during the diabetes development and NaHS enhanced TRPC6 protein expression in diabetic rats. In line with these results, we found that SKF-96365 treatment down-regulated TRPC6 protein expression in diabetic rats, whereas NaHS partially prevented the SKF-96365-induced diminution of the TRPC6 protein expression. Although data suggest for the first time the participation of TRPC channels in the H_2_S-induced hyperalgesia in diabetic rats, further experiments are necessary to clarify the mechanism by H_2_S activates/sensitizes TRPC channels. We decided to evaluate the protein expression of TRPC6, as a representative of TRP channels, because previous studies show that TRPC6 is present in DRG sensory neurons and it mediates hyperalgesia and nociceptor sensitization in normoglycemic animals [51, 52]. Thus, our results seem to indicate for the first time that TRPA1, TRPV1 and TRPC channels may function as molecular targets of H_2_S to maintain the hyperalgesia in diabetic rats.

H_2_S is produced from l-cysteine by enzymes CBS and CSE. Both enzymes have been involved in the processing of neuropathic pain [11, 12, 16–18]. In the current study we decided to investigate CBS enzyme for the following reasons: Syhr and colleagues reported that CSE is not critically involved in the processing of neuropathic pain [15] and we did not find any CSE signal with two commercial anti-CSE antibodies in DRG from normoglycemic or diabetic rats. On the contrary, we found that CBS was up-regulated during the diabetes development and its expression was increased and decreased by daily NaHS and hydroxylamine treatment, respectively, in diabetic rats. CBS protein expression correlated with chemical nociception as NaHS increased and hydroxylamine diminished formalin-induced flinching behavior in diabetic rats. That was important in order to exclude other possible mechanisms since hydroxylamine also increases the nitric oxide levels [53], activates A1 receptors [54] and ATP-sensitive potassium channels [55].

Double immunostaining of CBS with neuronal markers showed that CBS was expressed in about 60% of L4-L6 DRG neurons. This expression was mainly found in small- and medium-size cells. Our results are consistent with its localization on non-peptidergic neurons [current study] and with those studies performed in trigeminal, and L1 and S1 DRG neurons [15, 56] where CBS stains small- and medium-neurons with large DRG neurons staining weakly or absent. In addition, it has been reported that CBS is expressed in about 85% of L1 and S1 DRG neurons [15] and 77% of neurons from trigeminal ganglion [56]. Our results extend these observations to L4-L6 DRG. Moreover, CBS was highly co-localized with TRPV1, TRPA1 and TRPC6-positive neurons. These results confirm the co-expression of CBS with TRPV1 [56] or TRPA1 channels in sensory neurons [47] and, demonstrate for the first time the co-localization between CBS and TRPC6 in DRG from diabetic rats.

Repeated subcutaneous injection of NaHS not only enhanced streptozotocin-induced hyperalgesia, but also increased intraepidermal fiber loss in diabetic rats. Contrariwise, daily injection of hydroxylamine prevented the diabetes-induced intraepidermal nerve fiber loss. Results seem to indicate that H_2_S directly or indirectly activates TRP channels, which in turn, increase intracellular calcium inducing hyperalgesia and subsequent nerve fiber loss. Our results are in line with previous studies that point out that the loss of intraepidermal nerve fibers play a critical role in the development of various neuropathies, including diabetes [57, 58].

## Conclusions

In summary, our results suggest that H_2_S, synthetized by the CBS enzyme, activates/sensitizes peripheral TRPV1, TRPA1 and TRPC channels in non-peptidergic fibers to contribute to the streptozotocin-induced hyperalgesia and peripheral nerve fiber loss. In addition, data suggest that CBS, TRPA1, TRPV1 and TRPC channels could be potential targets for the treatment of peripheral neuropathy associated with diabetes.

## Methods

### Animals

Adult female Wistar rats weighing between 200 and 220 g were used in all experiments. Female rats were used based on the fact that there are no differences in formalin test between female and male rats in our conditions (Additional file 1: Figure S1). Animals were maintained in an air-conditioned room at 23–25 °C on a 12-h light/dark cycle, with ad libitum access to food and water and they were obtained from our own breeding facilities. All efforts were made to minimize the number of animals and their suffering.

### Drugs

*N*-[2-(4-Chlorophenyl)ethyl]-1,3,4,5-tetrahydro-7,8-dihydroxy-2*H*-2-benzazepine-2-carbothioamide (**capsazepine**), 1,2,3,6-tetrahydro-1,3-dimethyl-*N*-[4-(1-methylethyl)phenyl]-2,6-dioxo-7*H*-purine-7-acetamide (**HC-030031**), 1-[β-(3-(4-methoxyphenyl)propoxy)-4-methoxyphenethyl]-1*H*-imidazole hydrochloride (**SKF-96365**), sodium hydrosulfide (**NaHS**) and **hydroxylamine hydrochloride** were purchased from Sigma-Aldrich (St. Louis, MO, USA). All drugs were dissolved in isotonic saline solution with 10% of **dimethyl sulfoxide** and were freshly prepared the day of the experiment. All drug doses were selected from previous reports and pilot experiments in our conditions [18, 59–61].

### Streptozotocin-induced diabetes in rats

Experimental diabetes in rats was induced by a single intraperitoneal injection of streptozotocin (60 mg/kg), whereas control animals received an intraperitoneal injection of distilled water (1 mL/100 g of body weight). Diabetes was confirmed 3 days and 3 weeks after streptozotocin injection by measuring the tail vein blood glucose levels with a glucometer (FreeStyle Lite; Abbott Laboratories, Madrid, Spain). Rats with a blood glucose level ≥ 300 mg/dL were considered as diabetic and included in the study [18].

### Assessment of hyperalgesia in diabetic rats

Three weeks after streptozotocin injection, diabetic rats were placed in individual open acrylic observation chambers for an acclimation period of at least 30 min before testing. Then, they were removed and gently restrained while the dorsum of the hind paw was injected with 50 μL of 0.5% formalin (Jackson T. Barker, Pennsylvania, USA) using a 30-gauge needle. The animals were returned to the chambers and the flinching behavior was counted immediately. Nociceptive behavior was quantified as the number of flinches of the injected paw during 1-min periods every 5 min, up to 60 min after subcutaneous formalin injection [18, 62]. Flinching was easily discriminated and it was characterized as rapid and brief withdrawal or as flexing of the injected paw. Formalin-induced flinching behavior was biphasic. The first phase (0–10 min) was followed by a relatively short quiescent period, which was then followed by a prolonged second phase (15–60 min). The animals were used only once and were sacrificed in a CO_2_ chamber after the experiment. For euthanasia, rats were placed in a 36 L chamber and a 10 L/min CO_2_ flow rate was injected until rodents show lack of respiration and faded eye color (about 3 min). Formalin test was used to determine the possible molecular mechanisms underlying H_2_S-induced hyperalgesia in diabetic rats since this model is widely used to investigate painful diabetic neuropathy [63].

### Western blotting

Lumbar DRG (L4-L6) were excised and homogenized in 300 µL of ice-cold RIPA buffer containing 150 mM of NaCl, 50 mM of Tris–HCl, 1 mM of EDTA, 1% of tergitol-type NP-40, 0.1% of sodium dodecyl sulfate, 6.8 μg/mL of aprotinin, 4 μg/mL of leupeptin, 4 μg/mL of pepstatin A, 4 µg/mL of soybean trypsin inhibitor, 2 mM of phenylmethylsulfonyl fluoride and 2 mM of NaVO_4_ (Sigma-Aldrich, St. Louis, MO, USA). Then, homogenates were centrifuged at 14,000 rpm during 10 min at 4 °C. Supernatants were collected and the total protein concentration for each supernatant was determined by the Lowry’s method [64]. One hundred micrograms of total protein were loaded of each sample and they were resolved by 10% SDS–polyacrylamide gel electrophoresis. Proteins were transferred to polyvinylidene fluoride (PVDF) membranes. The membranes were blocked with 5% non-fat milk in phosphate-buffered saline at pH 7.4 containing 137 mM of NaCl, 2.7 mM of KCl, 10 mM of Na_2_HPO_4_ and 2 mM of KH_2_PO_4_ with 0.05% of Tween. After that, membranes were incubated overnight at 4 °C with the primary antibodies directed against the CBS (goat anti-CBS [K-14], Cat. # SC-46830, 1:200, Santa Cruz Biotechnology, TX, USA), TRPV1 (rabbit anti-TRPV1, Cat. # NB100-1617, 1:600, Novus Biologicals, CO, USA), TRPA1 (rabbit anti-TRPA1, Cat. # OST00061W, 1:800, Thermo Fisher Scientific, IL, USA) or TRPC6 (rabbit anti-TRPC6, Cat. # ab101622, 1:50, Novus Abcam, Cambridge, UK). Then, a horseradish peroxidase-conjugated secondary antibody (anti-goat, Cat. # 705-035-003, 1:10,000 or anti-rabbit, Cat. # 711-035-152, 1:6000, Jackson ImmunoResearch Laboratories Inc, PA, USA) was applied for detecting the respective primary antibody signal using an enhanced chemiluminescence detection system (Western Lightning Ultra, NEL112001EA, PerkinElmer, Waltham, MA, USA) with an imager (ChemiDoc ™ XRS + ImagingSystem, Bio-Rad, Hercules, CA, USA). After CBS, TRPV1, TRPA1 or TRPC6 detection, the membranes were stripped, blocked and incubated with a primary antibody directed against β-actin (mouse anti-actin, Cat. # 5512, 1:10,000, Gene-Tex, CA, USA) and its respective horseradish peroxidase-conjugated secondary antibody (anti-mouse, Cat. # 115-035-003, 1:3000, Jackson ImmunoResearch Laboratories Inc, PA, USA). β-actin was used as loading control to normalize the protein expression levels. For β-actin, was searched a single band of about 43 kDa. In addition, control peptide for CBS was used to determine the signal specificity. The densitometric analysis of the bands was performed with the ImageLab 5.0 software (Bio-Rad, Hercules, CA, USA). Bands of about 60 kDa, 95 kDa, 128 kDa and 107 kDa, corresponding to the CBS enzyme or TRPV1, TRPA1 and TRPC6 channels were searched in the ipsilateral L4–L6 DRG on normoglycemic and diabetic rats.

### Immunofluorescence

Rats were deeply anesthetized with isoflurane and then a thoracotomy was performed to expose the heart. A cannula was inserted into the ascending aorta through the left ventricle. After that, the right atrium was cut, and the rats were subjected to a cardiac perfusion with 250 mL of an ice-cold phosphate-buffered saline solution at pH 7.4 containing 137 mM of NaCl, 2.7 mM of KCl, 10 mM of Na_2_HPO_4_, 2 mM of KH_2_PO_4_ and 10 U/mL of heparin. Subsequently, perfusion solution was changed to 250 mL of an ice-cold phosphate-buffered saline solution at pH 7.4 containing 137 mM of NaCl, 2.7 mM of KCl, 10 mM of Na_2_HPO_4_, 2 mM of KH_2_PO_4_, 4% of paraformaldehyde and 12% of picric acid. Then, a laminectomy was performed and the L4–L6 DRG and hind paws were removed and placed in 20% sucrose solution during 72 h for cryoprotection. Later, L4–L6 DRG were embedded in a freezing medium (Tissue-Tek O.C.T. compound, Sakura Finetek, CA, USA) and cut by a cryostat (Leica CM1950, Leica Biosystems, Wetzlar, Germany) at − 25 °C. Longitudinal cross-sections of 12 µm were made from DRG. The cross-sections were received in gelatinized glass slides and held at − 20 °C until use. On the test day, the gelatinized glass slides containing the tissues were washed three times in phosphate-buffered saline solution at pH 7.4 for 10 min each and the tissue area in each glass slide was delimited with a hydrophobic slide marker (Pap-pen, BioGenex, CA, USA). Next, tissues were blocked with 150 μL of phosphate-buffered saline at pH 7.4 containing 10% of normal donkey serum and 0.3% of triton X-100 for 1 h. After the blocking time, the solution was changed to 150 μL of phosphate-buffered saline at pH 7.4 containing 1% of normal donkey serum, 0.1% of triton X-100 and the primary antibody against CBS (goat anti-CBS [K-14], Cat. # SC-46830, 1:200, Santa Cruz Biotechnology, TX, USA) in combination with the primary antibody against one of the following channels: TRPV1 (rabbit anti-TRPV1, Cat. # NB100-1617, 1:600, Novus Biologicals, CO, USA), TRPA1 (rabbit anti-TRPA1, Cat. # OST00061W, 1:800, Thermo Fisher Scientific, IL, USA) and TRPC6 (rabbit anti-TRPC6, Cat. # ab101622, 1:50, Novus Abcam, Cambridge, UK) or in combination with the one of the following cellular marker: neurons (mouse anti-neuronal nuclear antigen [NeuN], Cat. # MAB377, 1:200, Millipore, MA, USA), peptidergic neurons (guinea pig anti-substance P [SP], Cat. # GP14110, 1:200, Neuromics, MN), non-peptidergic neurons (biotinylated-isolectin IB4, Cat. # L2140, 1:500, Sigma-Aldrich, MO, USA) and astrocytes (mouse anti-glial fibrillary acidic protein [GFAP], Cat. # G3893, 1:500, Sigma-Aldrich, MO, USA). Tissues were incubated overnight at 4 °C. Then, they were washed three times in phosphate-buffered saline solution at pH 7.4 for 10 min each and 150 μL of phosphate-buffered saline at pH 7.4 containing 1% of normal donkey serum, 0.1% of triton X-100 and the respective Cy3 and Cy2 fluorophore-conjugated secondary antibodies (Cy3 anti-goat, Cat. # 705-165-003, 1:600 or Cy3 anti-rabbit, Cat. # 711-166-152, 1:600; and Cy2 anti-rabbit, Cat. # 711-225-152, 1:200 or Cy2 anti-mouse, Cat. # 715-225-148, 1:200, or Cy2 anti-guinea pig, Cat. # 706-225-148, 1:200, or Cy2-streptavidin, Cat. # 016-220-084, 1:600, Jackson ImmunoResearch Laboratories Inc, PA, USA). Again, tissue sections were incubated for 1.5 h at room temperature and they were washed three times in phosphate-buffered saline solution at pH 7.4 for 10 min each. After that, sections were dehydrated through 70%, 80% 90% and 100% ethanol and twice xylol for 2 min each. Sections were mounted in 100 μL of DPX mounting medium (Sigma-Aldrich, MO, USA) and coated with an anti-fading coverslip (Gold Seal, Thermo Scientific, MA, USA). The lamellae were observed under a microscope (Nikon Eclipse Ni, Nikon Corporation, Tokyo) coupled to fluorescence (C-HGFI, Nikon Corporation, Tokyo, Japan). Images were captured with a 5.1 MP camera (Evolution MP color, Media Cybernetics, MD, USA) and analyzed by the software Image-Pro Premier 9.1 (Media Cybernetics, MD, USA).

### Immunohistochemistry for paraffin-embedded tissue

Rats were deeply anesthetized with isoflurane as was above described. 3–4 mm biopsies of the glabrous skin from the footpad of the right paw were obtained and, the tissues were fixated in formaldehyde during 24 h, after that, they were dehydrated through 70%, 80% 90% and three times 100% ethanol and twice xylol for 10 min each, using a tissue processor (Thermo Scientific™ Citadel 2000, MA, USA). Then, biopsies were embedded twice in paraffin for 1.5 h each (Shandon Histocentre 2, MA, USA). Longitudinal cross-sections of 5 µm were made from hindpaw biopsies by a microtome (Leica RM2235, Leica Biosystems, Wetzlar, Germany), the cross-sections were received in adhesion glass slides (Millennia 2.0, StatLab, TX, USA) and held at room temperature until use. On the test day, the hindpaw tissues sections were deparaffinized at 55 °C for 30 min and rehydrated through twice xylol, twice absolute ethanol and, once 90%, 80% and 70% ethanol and water for 2 min each. Next, they were washed twice with 20 mL of an ice-cold tris buffered saline solution at pH 7.4 (TBST, containing 20 mM of Tris base, 150 mM of NaCl and 0.1% Tween 20) for 10 min each. The tissue area in each glass slide was delimited with a hydrophobic slide marker (Pap-pen, BioGenex, CA, USA). Then, tissues were submitted to the pressure cooker for the heat-induced epitope retrieval in a sodium citrate buffer at pH 6.0 (containing 10 mM sodium citrate and 0.5% tween 20) during 3 min. After that, tissues were blocked with a methanol solution containing 2% hydrogen peroxide for 30 min, washed three times in TBST for 10 min each and, blocked again with TBST (containing 1% bovine serum albumin, 10% horse normal serum and 0.1% saponin) at room temperature for 2 h. After the blocking time, the primary antibody (rabbit-anti PGP 9.5, Cat. # 14730-1-AP, 1:50, Proteintech, IL, USA) diluted in TBST with 1% bovine serum albumin and 1.5% horse normal serum was applied in the tissues for 90 min. Next, slides were washed three times in TBST for 10 min each and, the tissues were exposed with a secondary biotinylated antibody for 60 min and incubated with avidin-biotinylated horseradish peroxidase complex for 60 min according to the manufacturer’s instructions (Vectastain ABC kit for Rabbit IgG, Cat. # PK-6101, Vector Laboratories, CA, USA). Color development was accomplished with 3,3′-diaminobenzidine for 3 min (Liquid DAB + Substrate Chromogen System, Cat. # K3467, Agilent, CA, USA) and the slides were washed three times with distilled water for 10 min each. Immediately, tissues were counterstained with a Harris hematoxylin for 30 s and dehydrated through twice distilled water, three times 0.25% ammonia water, 70%, 80% 90% and 100% ethanol and twice xylol for 30 s each. Section were mounted in 100 μL of DPX mounting medium (Sigma-Aldrich, MO, USA) and coated with an anti-fading coverslip (Gold Seal, Thermo Scientific, MA, USA). The tissues were observed under a microscope (Leica DM750, Leica Biosystems, Wetzlar, Germany). Images were captured with a 5 MP camera (Leica ICC50 E, Leica Biosystems, Wetzlar, Germany) and analyzed by the software Image-Pro Premier 9.1 (Media Cybernetics, MD, USA). To obtain the PGP 9.5 immunodensity, we counted all immunoreactive profiles within the epidermis and subepidermal nerve plexus. Epidermal nerve fibers were counted and normalized to an average of 3 mm of epidermal length.

### Study design

Independent groups of six rats were used for all behavioral experiments. In order to determine the effect of experimental diabetes on nociception, we used the formalin test in normoglycemic rats injected with vehicle or diabetic rats injected with streptozotocin injection (60 mg/kg, i.p.) at 7, 14 and 21 days. To evaluate sex differences in our behavioral model, we compared the flinching behavior between female and male diabetic rats at 21 days post-streptozotocin injection in the 0.5% formalin-induced flinching behavior. To investigate the role of peripheral H_2_S in diabetes-induced hyperalgesia, we carried out a dose–response curve to NaHS in diabetic rats (3 weeks). For this, we administered vehicle or increasing doses of NaHS (3–100 µg/paw, sc) 10 min before 0.5% formalin injection. As control, we injected NaHS 100 µg/paw into the contralateral paw. Furthermore, in order to evaluate the participation of peripheral TRP channels in diabetic rats, we injected vehicle or increasing doses of capsazepine (non-selective TRPV1 blocker, 0.3–30 µg/paw, sc), HC-030031 (selective TRPA1 blocker, 100–1000 µg/paw, sc) and SKF-96365 (non-selective TRPC blocker, 10–100 µg/paw, sc) 20 min before 0.5% formalin injection into the ipsilateral paw. In an attempt to establish whether NaHS-induced hyperalgesia was mediated by TRPA1, TRPV1 or TRPC activation in diabetic rats (3 weeks), we co-administered the highest dose tested of NaHS (100 µg/paw, sc, − 10 min) with increasing ineffective doses of capsazepine (0.3 and 3 µg/paw, sc, − 20 min), HC-030031 (100 and 316 µg/paw, sc, − 20 min) or SKF-96365 (10 and 30 µg/paw, sc, − 20 min) in 0.5% formalin-treated diabetic rats. To determine whether drugs acted locally, the highest tested doses of drugs were administered individually to the left paw (contralateral) whereas 0.5% formalin was injected into the right paw (ipsilateral), and the nociceptive behavior was assessed. Formalin at 0.5% was used in diabetic rats due this concentration let us determine in this metabolic condition pro- and antinociceptive effects, as was previously validated [18].

Independent groups of 3–6 rats were used for each experiment in all molecular and immunohistochemistry studies. In order to determine if experimental diabetes modulated expression of CBS, TRPV1, TRPA1 and TRPC6, streptozotocin-induced diabetic rats were sacrificed at 21 days to harvest DRG. CBS, TRPV1, TRPA1 and TRPC6 protein levels were determined by western blot. Since experimental diabetes enhanced CBS and TRPC6 expression, we decided to determine the expression of CBS and TRPC6 protein expression levels in presence of the H_2_S-producing enzyme CBS inhibitor hydroxylamine (CBS inhibitor, 0.1 µg/paw, s.c., daily for 21 days) and the exogenous H_2_S donor NaHS (100 µg/paw, s.c., daily for 21 days). Hydroxylamine dose was chosen on the basis of a previous study in our conditions [18]. In addition, TRPC6 expression was measured in presence of the TRPC6 inhibitor SKF-96365 (100 μg/paw, s.c., daily for 21 days) alone or co-administered with NaHS (100 µg/paw, s.c., daily for 21 days). TRPA1, TRPV1 and TRPC6 signal specificity was characterized by eliminating the primary antibody, whereas CBS signal specificity was tested using its control antigenic peptide.

In order to determine the specific cellular and subcellular distribution of the CBS enzyme in lumbar DRG, we performed double staining with the following markers: NeuN was used as a specific neuronal marker; SP was used as a specific marker of peptidergic neurons; IB4 was used to label non-peptidergic neurons; and GFAP was used to identify astrocytes. Considering that the distribution of CBS-positive neurons in normoglycemic and diabetic rats was similar, double immunostaining was carried out in DRG of diabetic rats to establish co-localization of CBS with TRPV1, TRPA1 and TRPC6 channels. TRPA1, TRPV1 and TRPC6 signal specificity was tested eliminating the primary antibody, whereas CBS signal specificity was determined using a mixture of CBS + peptide (1:1) on IB4-positive neurons. In order to stablish the effect of H_2_S on cutaneous innervation in the rat hindpaw, we carried out immunostaining with PGP 9.5, as a marker of peripheral nerve fibers, in normoglycemic and diabetic rats treated with vehicle, NaHS or hydroxylamine during 21 days.

### Data analysis and statistics

All behavioral data are presented as the mean ± SEM of 6 animals per group. Time courses were constructed by plotting the number of paw flinches as a function of time. Dose–response curves were obtained by the area under the curve calculated from time courses by the trapezoidal rule, as was previously described [18]. Student’s *t* test or one-way analysis of variance (ANOVA) followed by the Dunnett’s or Tukey’s test was used to compare differences between treatments.

Bar plots of protein expression and number of fibers are presented as the mean ± SEM of 3–6 independent experiments. Student’s *t* test or one-way ANOVA followed by the Student–Newman–Keuls’ test was used to compare differences between treatments. Finally, the bars in the distribution histograms are presented as the mean ± SEM of 10–12 longitudinal cross-sections from three rats. Two-way ANOVA test was used to compare differences between CBS-positive neurons of normoglycemic and diabetic rats. In all cases, differences were considered statistically significant when *P* < 0.05.

## Additional file

**Additional file 1: Figure S1.** Formalin-induced nociceptive behavior in female and male diabetic rats. (**A**) Time course of the nociceptive behavior observed after injection of 0.5% formalin into the dorsal hind paw of female and male diabetic rats. (**B**) Nociceptive behavior induced by 0.5% formalin during first and second phase in female and male diabetic rats. Data are expressed as mean ± SEM for 6 animals. There is no significant difference between female and male diabetic rats, determined by *t* Student test.

## Abbreviations

ANOVA: analysis of variance
BCTC: 4-(3-chloro-2-pyridinyl)-*N*-[4-(1,1-dimethylethyl)phenyl]-1-piperazinecarboxamide
CBS: cystathionine-β-synthase
CREB: cAMP response element-binding
CSE: cystathionin-γ-lyase
DRG: dorsal root ganglia
ERK1/2: extracellular signal-regulated protein kinases 1 and 2
GFAP: glial fibrillary acidic protein
H_2_S: hydrogen sulfide
HA: hydroxylamine
HC-030031: 1,2,3,6-tetrahydro-1,3-dimethyl-*N*-[4-(1-methylethyl)phenyl]-2,6-dioxo-7*H*-purine-7-acetamide
IB4: isolectin B4
kDa: kilodaltons
NaHS: sodium hydrosulfide
NeuN: neuronal nuclear antigen
NF-κβ(p65): subunit p65 of NF-kappa-B transcription complex
SEM: standard error of the mean
SD: standard deviation
SKF-96365: 1-[β-(3-(4-Methoxyphenyl)propoxy)-4-methoxyphenethyl]-1*H*-imidazole hydrochloride
SP: substance P
STZ: streptozotocin
TRP: transient receptor potential
TRPA1: transient receptor potential ankyrin 1
TRPV1: transient receptor potential cation channel vanilloid 1
TRPC: transient receptor potential canonical
TRPC6: transient receptor potential canonical 6
